# Safety and efficacy of intravenous tPA after successful thrombectomy for large vessel occlusion: a retrospective study

**DOI:** 10.3389/fneur.2026.1793073

**Published:** 2026-05-29

**Authors:** Masashi Kotsugi, Tomoya Okamoto, Hiromichi Hayami, Kenta Nakase, Yudai Morisaki, Shohei Yokoyama, Ryosuke Matsuda, Shuichi Yamada, Ichiro Nakagawa

**Affiliations:** Department of Neurosurgery, Nara Medical University, Nara, Japan

**Keywords:** acute ischemic stroke, endovascular thrombectomy, intra-venous thrombolysis, large vessel occlusion, parenchymal blood volume

## Abstract

**Introduction:**

This study aimed to evaluate the efficacy and safety of adjunctive intravenous (IV) tissue plasminogen activator (tPA) administration following successful mechanical thrombectomy in patients with acute ischemic stroke caused by large vessel occlusion (LVO).

**Methods:**

This single-center retrospective study included patients with anterior circulation LVO who achieved successful recanalization (Thrombolysis in Cerebral Infarction score ≥2b) after endovascular thrombectomy (EVT) between January 2021 and December 2024. Beginning in December 2022, 25% of the standard IV tPA dose (0.6 mg/kg) was administered immediately after successful recanalization (post-IV tPA group), in addition to the standard pre-EVT 75% dose. Parenchymal blood volume (PBV) ratios were assessed using flat-panel detector computed tomography before and after EVT. The primary outcome was the modified Rankin Scale (mRS) score at 90 days. Safety outcomes were symptomatic intracranial hemorrhage (sICH), any intracranial hemorrhage (ICH), and 90-day mortality.

**Results:**

Fifty-five patients met inclusion criteria (post-IV tPA, *n* = 21; standard care, *n* = 34). The post-IV tPA group showed significantly higher post-EVT PBV ratios (1.45 vs. 1.20, *p* = 0.03) and greater ΔPBV (2.45 vs. 1.81, *p* = 0.011). Good functional outcomes (mRS 0–2 at 90 days) were more frequent in the post-IV tPA group (61.9% vs. 29.4%, *p* = 0.025). No significant differences were seen in overall ICH (14.3% vs. 14.7%, *p* = 1.00), sICH (9.5% vs. 8.8%, *p* = 1.00), or 90-day mortality (0% vs. 9%, *p* = 0.54). Patients who exhibited lesion growth on diffusion-weighted imaging showed significantly lower post-EVT PBV and ΔPBV ratios (*p* = 0.002 and 0.0134, respectively). In subgroup analysis, longer onset-to-recanalization time was associated with hemorrhagic events in the post-IV tPA group (307 min vs. 226 min, *p* = 0.04).

**Conclusion:**

Adjunctive IV tPA administration following successful EVT may improve microvascular reperfusion and functional outcomes without increasing hemorrhagic complications, although careful patient selection is warranted. Due to study limitations, results are hypothesis-generating and require further validation in future prospective randomized trials.

## Introduction

Endovascular thrombectomy (EVT) is the current standard treatment for patients with acute ischemic stroke due to large vessel occlusion (LVO), offering high rates of recanalization (1). However, favorable clinical outcomes remain suboptimal, with many patients experiencing poor recovery despite technically successful reperfusion (2, 3). This discrepancy has been partly attributed to the no-reflow phenomenon, in which macrovascular recanalization does not result in adequate microvascular reperfusion. In addition, residual thrombotic occlusions in distal arteries may hinder complete tissue-level perfusion (4). Recent perfusion imaging studies have revealed that microcirculatory impairment, reflected by reduced cerebral blood flow and volume, can persist even after EVT and is associated with infarct growth and poor outcomes (5). The underlying mechanisms may involve distal microthrombi, endothelial dysfunction, and capillary blockage, all of which contribute to inadequate tissue-level perfusion. Adjunctive pharmacological strategies targeting this microvascular deficit are gaining attention. Intra-arterial thrombolysis (IAT) after EVT has shown promise in improving functional outcomes at 90 days without significantly increasing hemorrhagic complications (6, 7). However, the potential of intravenous (IV) administration of low-dose tissue plasminogen activator (tPA) after thrombectomy remains largely unexplored. IAT has been investigated as a means to improve microvascular reperfusion, but requires re-navigating a catheter into already recanalized vessels, which may increase the risk of vascular injury and require advanced endovascular skills. In contrast, IV administration is technically simpler, avoids further endovascular manipulation, and can be seamlessly integrated into the post-thrombectomy treatment process. These advantages make IV post-thrombectomy tPA an attractive and potentially safer strategy to address residual microvascular perfusion deficits. This retrospective study investigated whether IV administration of a low dose of tPA following successful mechanical thrombectomy can enhance microvascular perfusion and improve functional outcomes in patients with LVO-related acute ischemic stroke.

## Methods

This study is a retrospective analysis of prospectively collected data from our institutional stroke registry. The study was conducted in accordance with the Strengthening the Reporting of Observational Studies in Epidemiology (STROBE) statement. The institutional review board of our hospital approved all the study protocols (approval no. 2370).

### Inclusion criteria

All patients with anterior circulation LVO who underwent mechanical thrombectomy at our institution between January 2021 and December 2024 were considered for inclusion in this study. Patients with a Thrombolysis in Cerebral Infarction (TICI) score >2b were selected for analysis. The standard method group received 100% of the standard IV-tPA dose prior to EVT. Beginning in December 2022, patients received 75% of the standard dose of IV tPA (0.6 mg/kg) according to the standard protocol prior to thrombectomy (post-IV tPA group). The remaining 25% of the dose was administered intravenously immediately after confirmation of successful recanalization. The decision to administer adjunctive post-IV tPA was not randomized but rather based on the evolution of our institutional treatment protocol over time. Our institution adopted a modified protocol wherein 75% of the standard tPA dose was administered initially and 25% was reserved for immediate post-thrombectomy administration, aiming to improve microvascular reperfusion while avoiding the increased hemorrhagic risk associated with exceeding the standard total dose. Furthermore, all patients who received IV-tPA (both the standard 100% pre-EVT dose and the 75–25% split dose) were administered the drug within the standard therapeutic window of 4.5 h from symptom onset, in accordance with current guidelines. The concept of a quarter-dose adjunct is consistent with the CHOICE randomized trial, which administered intra-arterial alteplase at 25% of the standard dose after successful thrombectomy and showed improved functional outcomes without excess symptomatic intracranial hemorrhage (sICH) (6). Although the route of administration in CHOICE was intra-arterial rather than IV, the trial provides supporting evidence for the biological plausibility and safety of a quarter-dose alteplase supplement in the post-thrombectomy setting. Patients for whom thrombectomy was completed during the infusion of the initial 75% dose of IV tPA were excluded.

Parenchymal blood volume (PBV) was measured using flat-panel detector computed tomography (FD-CT) before and after procedures. Considering the half-life of tPA, PBV was performed 15 min after the completion of IV tPA following EVT. The region of interest (ROI) included six brain regions, and PBV was calculated as the PBV on the ischemic side divided by that on the contralateral side (the measurement method is described in detail in the ‘PBV measurement’ section). Baseline clinical characteristics recorded for each patient included age, sex, history of risk factors, and preoperative National Institutes of Health Stroke Scale (NIHSS) score. Hyperlipidemia was defined as a serum low-density lipoprotein cholesterol level ≥ 120 mg/dL. The primary outcome was the functional outcome at 90 days, assessed using the modified Rankin Scale (mRS), with scores of 0–2 considered a good outcome. Secondary outcomes included the change in the PBV ratio after treatment and the mRS score at discharge. Safety outcomes included sICH, any intracranial hemorrhage (ICH), and all-cause mortality at 90 days. ICH and sICH were evaluated based on follow-up CT or MRI scans performed routinely at 24–48 h post-procedure, or earlier if neurological deterioration occurred.

### PBV measurement

PBV assessment in stroke patients shows a correlation with cerebral blood volume (CBV) and demonstrates reasonable sensitivity and specificity for detecting hypoperfused regions (8, 9). FD-CT was performed before and after EVT using a biplane FD angiographic system (Axiom Artis Q; Siemens Healthineers, Erlangen, Germany) under sedation with dexmedetomidine. Image acquisition included two separate 200° rotational scans: the first was a non-contrast (mask) run, followed by a contrast-enhanced (fill) run performed via a 4-Fr pigtail catheter positioned above the aortic valve. To ensure that the fill run was acquired during the steady-state phase of contrast enhancement in the brain parenchyma, the C-arm was returned to its original starting position after the mask run. Standard two-dimensional digital subtraction angiography (DSA) was then performed at a rate of 2 frames per second. Dilute contrast medium (50%) was injected at 5.0 mL/s and the fill run was then started manually when opacification of the venous sinus was observed by the operator.

Acquisition time for both mask and fill runs was 6.6 s, with 2-dimensional projections acquired at a rate of 60 frames/s at 70 kV, with a matrix size of 616 × 480. Postprocessing of FD perfusion was carried out using Syngo DynaPBV Neuro on a Syngo X Workplace, version VD11C (Siemens Healthineers). For PBV image processing, subtracted data were normalized with an input function automatically estimated from histographic analysis of the vascular tree. We then performed color-coding with a standard color table to generate PBV color-coded maps for visualization and interpretation. A circular ROI with a diameter of 10 mm was then positioned on the homogeneous area excluding the cerebral cortex and avoiding volume-averaging with the adjacent sulcus. PBV values were derived from 6 ROIs of periventricular, deep white matter, and hemispheric center on three slices. As a control, PBV values were measured in an equivalent slice location mirrored in the contralateral hemisphere. PBV ratio was calculated as the average of PBV values/equivalent slice PBV values mirrored in the contralateral hemisphere. PBV change was calculated as the average of PBV values in the affected hemisphere after EVT/the average of PBV values before EVT.

### Diffusion-weighted imaging (DWI) assessment

Lesion growth on DWI was assessed by comparing baseline DWI with follow-up DWI acquired 24–72 h after EVT. Two independent neuroradiologists blinded to treatment allocation visually classified each case as “growth” (new or clearly enlarged DWI lesion on follow-up) or “no growth.” Discrepancies were resolved by consensus with a third reader.

### Interventions

All procedures were performed under local anesthesia. Unfractionated heparin was not administered during the procedure in any of the cases. A 9-Fr balloon guiding catheter was advanced into the cervical internal carotid artery (ICA).

EVT was performed at the discretion of the operator using either an aspiration catheter alone (A direct aspiration first pass technique (ADAPT) technique) or a combined technique employing both a stent retriever and an aspiration catheter. The specific devices used were also selected at the discretion of the operator. In the post-IV tPA group, patients received 75% of the standard dose of IV tPA (0.6 mg/kg) according to the institutional protocol. The remaining 25% of the dose was administered intravenously immediately after successful recanalization was confirmed.

### Statistical analysis

Continuous variables showing normal distributions are presented as mean ± standard deviation and were compared between the two study periods using Student’s *t*-test, Fisher’s exact tests and analysis of variance. Non-parametric continuous variables are presented as median and interquartile range (IQR) and were compared with a Wilcoxon matched-pair signed-rank test for paired samples or the Mann–Whitney U test for independent samples. Variables showing values of *p* < 0.15 in univariate analyses were entered into a multivariate logistic regression model. Odds ratios with 95% confidence intervals were calculated. Differences were deemed statistically significant at the level of *p* < 0.05.

## Results

Between January 2021 and December 2024, a total of 212 patients underwent thrombectomy for LVO in our institute. Table 1 presents the clinical characteristics of the 55 enrolled patients (31 men, 24 women). Median ages of patients in the standard method group (*n* = 34) and post-IV tPA group (*n* = 21) were 81 years (IQR, 70.25–87 years) and 79 years (IQR, 74.5–85 years), respectively.

**Table 1:** Clinical characteristics of the 55 patients.

Treatment group	Standard method (*n* = 34)	Post-iv. tPA (*n* = 21)	*p*-value
General characteristics			
Median age (IQR)	81 (70.25–87)	79 (74.5–85)	0.67
Female	17 (50%)	7 (33%)	0.27
Pre-onset mRS			0.67
0	5	4	
1	29	17	
Risk factor			
Hypertension	24 (71%)	16 (76%)	0.76
Diabetes	10 (29%)	6 (29%)	1.00
Hyperlipidemia	14(41%)	11 (52%)	0.58
Etiology			0.35
Cardioembolism	28 (82%)	19 (90%)	
Atherosclerosis	4 (12%)	2(10%)	
Other	2 (6%)	0 (0%)	
Lesion location			0.79
ICA	10 (29%)	7 (33%)	
M1	13 (38%)	9 (43%)	
M2	11 (32%)	5 (24%)	
Baseline stroke severity			
NIHSS score on admission	19.7 ± 9.1	20.3 ± 10.1	0.83
DWI-ASPECTS	7.2 ± 2.1	7.8 ± 2.8	0.35

ICA, internal carotid artery; M1, M1 segment of the middle cerebral artery; M2, M2 segment of the middle cerebral artery; DWI, diffusion-weighted imaging.

### Baseline characteristics, risk factors, and etiology

**Figure 1:**
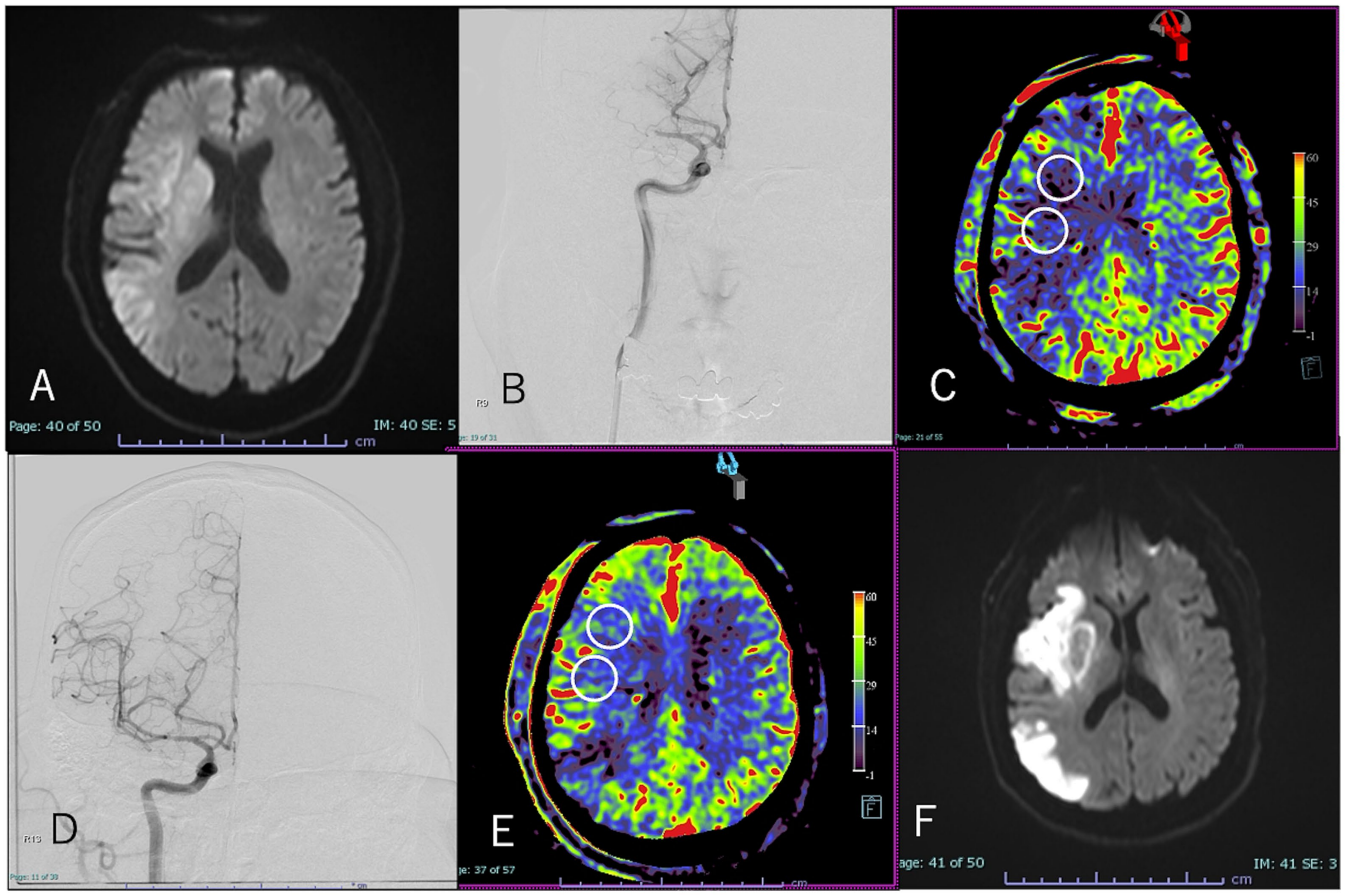
Representative case of poor parenchymal blood volume (PBV) reperfusion after endovascular thrombectomy (EVT). A 66-year-old patient presented with sudden onset of severe left hemiparesis and dysarthria. On admission, National Institutes of Health Stroke Scale (NIHSS) score was 18/42. **(A)** Magnetic resonance angiography (MRA) reveals occlusion of the right middle cerebral artery (MCA) and diffusion-weighted imaging (DWI) shows hyperintensity in the right MCA territory. **(B)** Digital subtraction angiography confirms right MCA occlusion. **(C)** Pre-EVT flat-panel detector computed tomography perfusion (FD-CTP) demonstrates hypoperfusion on PBV mapping in the right hemisphere (white circles; pre-PBV ratio, 0.81). **(D)** Complete angiographic recanalization (mTICI 3) was achieved with EVT (onset-to-recanalization time, 309 min). **(E)** However, post-EVT FD-CTP shows no meaningful increase in PBV in the affected region (white circles; post-PBV ratio, 1.13; ΔPBV, 1.40). **(F)** Follow-up DWI reveals extensive infarction in the right MCA territory.

Lesion location/stroke severity did not differ significantly between groups (Table 1). PBV parameters, including post-EVT PBV ratio and ΔPBV ratio (post/pre), were significantly higher in the post-IV tPA group compared with the standard group (*p* = 0.03 and *p* = 0.011, respectively). DWI analysis showed a trend toward a lower incidence of infarct growth in the post-IV tPA group (5 of 21, 23.8%) than in the standard group (16 of 34, 47.1%; *p* = 0.08). Further, to evaluate the relationship between cerebral perfusion and infarct progression, we compared PBV parameters according to the presence of DWI lesion growth. Patients who exhibited DWI lesion growth had significantly lower post-EVT PBV and ΔPBV ratios (*p* = 0.002 and *p* = 0.0134, respectively) (Supplementary Figure 1). Similarly, good efficacy outcomes (defined as mRS 0–1 at discharge and mRS 0–2 at 90 days) also differed significantly between groups (*p* = 0.03 and *p* = 0.025, respectively) (Table 2). There was no significant difference in the number of thrombectomy passes between the standard method group and the post-IV tPA group (1.88 ± 1.09 vs. 1.57 ± 1.08, *p* = 0.31). Similarly, there was no significant difference in the initiation time [onset-to-puncture (O2P)] between the two group (190.3 ± 51.1 min vs. 194.7 ± 63.4 min, *p* = 0.80). Additionally, while P2R was significantly shorter in the post-IV tPA group, the total onset-to-reperfusion (O2R) time did not differ significantly between the two groups (the standard group 261.2 ± 56.8 min. vs. the post-IV tPA group 239.6 ± 63.4 min., *p* = 0.24; Table 2). Regarding safety, no significant differences were observed between the two groups in terms of hemorrhagic complications or 90-day mortality (Table 2). In the overall cohort, patients who developed sICH tended to have lower ΔPBV and longer onset-to-recanalization (O2R) times, although these differences did not reach the level of statistical significance (ΔPBV: 1.54 vs. 2.19, *p* = 0.13; O2R: 293.2 min vs. 246.9 min, *p* = 0.10). However, within the post-IV tPA group, patients who experienced hemorrhagic events displayed significantly longer O2R times (307 min) compared to those who did not (226 min, *p* = 0.04). No significant association was observed between PBV parameters and hemorrhagic complications in this subgroup. A representative case of poor PBV reperfusion after angiographic recanalization by EVT is shown in Figure 1. Additionally, a subgroup analysis evaluating sex differences revealed no significant disparity between male and female patients regarding the rate of good efficacy outcomes (mRS 0–1 at discharge and mRS 0–2 at 90 days) (*p* = 0.56 and *p* = 0.57, respectively) and PBV parameters (post-EVT PBV and ΔPBV ratios (*p* = 0.96 and *p* = 0.31, respectively)).

**Table 2:** Procedural outcomes for the 55 patients.

Treatment group	Standard method (*n* = 34)	Post-i.v. tPA (*n* = 21)	*p*-value
Procedure			
The number of passes	1.88 ± 1.09	1.57 ± 1.08	0.31
O2R (min)	261.2 ± 56.8	239.6 ± 63.4	0.24
O2N (min)	157.2 ± 59.5	174.4 ± 52.0	0.33
O2P (min)	190.3 ± 51.1	194.7 ± 63.4	0.80
D2R (min)	148.6 ± 54.1	147.1 ± 57.6	0.93
P2R (min)	68.2 ± 29.3	44.9 ± 23.9	0.005
PBV			
Pre-EVT PBV ratio	0.70 ± 0.17	0.62 ± 0.18	0.22
Post-EVT PBV ratio	1.20 ± 0.32	1.45 ± 0.26	0.03
ΔPBV ratio (post/pre)	1.81 ± 0.68	2.45 ± 0.57	0.011
DWI			
Lesion growth	16 (47.1%)	5 (23.8%)	0.08
Efficacy outcome			
mRS 0–1 at discharge	6 (17.7%)	10 (47.6%)	0.03
mRS 0–2 at discharge	10 (29.4%)	12 (57.1%)	0.052
mRS 0–2 at 90 days	10 (29.4%)	13 (61.9%)	0.025
Safety outcome			
ICH	5 (14.7%)	3 (14.3%)	1.00
sICH	3 (8.8%)	2 (9.5%)	1.00
Mortality at 90 days	3 (9%)	0 (0%)	0.54

O2R, onset-to-recanalization time; O2N, onset-to-needle time; D2R, door-to-recanalization time; P2R, puncture-to-recanalization time; PBV, parenchymal blood volume; EVT, endovascular thrombectomy; ICH, intracranial hemorrhage; sICH, symptomatic intracranial hemorrhage.

## Discussion

This study suggests that IV tPA following successful mechanical thrombectomy may improve microvascular perfusion, as evidenced by increased post-EVT PBV and ΔPBV ratios. Importantly, patients who demonstrated greater improvement in PBV were less likely to show infarct expansion on follow-up DWI. Lesion growth on DWI was associated with significantly lower post-EVT PBV and ΔPBV ratios. These findings are compatible with the hypothesis that adjunctive IV tPA may help dissolve residual distal microthrombi and thereby mitigate the no-reflow phenomenon, resulting in improved tissue-level reperfusion and, ultimately, better functional outcomes.

EVT is the current standard treatment for patients with acute ischemic stroke caused by LVO (1). However, despite successful recanalization, some patients still experience poor clinical outcomes (2, 3). This discrepancy has been partly attributed to the no-reflow phenomenon, in which macrovascular recanalization does not result in adequate microvascular reperfusion, as well as to residual thrombotic occlusions in distal arteries, inflammatory processes and compression due to functional or structural change in the vessel wall or surrounding cells, all of which may hinder complete tissue-level perfusion (4, 10, 11). Recent perfusion imaging studies have revealed that microcirculatory impairment, as reflected by reduced cerebral blood flow and volume, can persist even after EVT and is associated with infarct growth and poor outcomes (12, 13). Our findings are consistent with these observations. In this study, post-EVT PBV ratio and ΔPBV were significantly higher in patients who received IV tPA after successful thrombectomy, compared to those who did not. These results suggest that additional low-dose tPA administration may improve downstream microvascular perfusion, possibly by dissolving residual microthrombi in the distal vasculature. This implies that adjunctive tPA could mitigate the no-reflow phenomenon and enhance tissue-level reperfusion, even in cases where macrovascular recanalization has been technically achieved.

Previous studies have established that successful reperfusion with fewer thrombectomy passes is strongly associated with favorable functional outcomes (14). In our study, however, there was no significant difference in the number of passes between the two groups. Crucially, while the post-IV tPA group demonstrated a significantly shorter P2R, O2R did not differ significantly between the cohorts. The equivalent O2R suggests that the improved ΔPBV and functional outcomes in the post-IV tPA group were not merely driven by faster procedural times. Instead, our findings highlight the pharmacological benefits of IV tPA. These pharmacological effects likely mitigated the no-reflow phenomenon, directly contributing to the favorable clinical outcomes and enhanced tissue-level perfusion observed in this group.

Despite these favorable pharmacological effects, the no-reflow phenomenon appears to be multifactorial (11). Potential mechanisms include pericyte-mediated capillary constriction, inflammatory plugging by neutrophils, and endothelial dysfunction, all of which can contribute to persistent microvascular obstruction and sustained tissue hypoperfusion. While tPA may alleviate thrombotic occlusions, this therapy is unlikely to directly address inflammatory or vasomotor components of no-reflow. This limitation underscores the need to explore combination strategies that target both thrombotic and non-thrombotic pathways of microvascular impairment.

Several previous studies have explored IAT after EVT as a strategy to improve microvascular reperfusion, with some reports suggesting improved functional outcomes without a significant increase in hemorrhagic complications (6, 7). However, IAT requires re-navigating a catheter into vessels that have already been successfully recanalized, which may increase the risk of vascular injury or other procedure-related complications. In contrast, our approach utilizes a simplified IV route, in which a portion of the standard tPA dose is administered immediately after reperfusion is achieved. This strategy avoids the need for additional catheter manipulation and may reduce procedural complexity and risk. Moreover, IV administration can be seamlessly integrated into the workflow without prolonging procedure time, and does not require precise catheter positioning, making the method less dependent on operator experience. Conversely, a potential disadvantage of the IV route compared to the IA route (as utilized in the CHOICE trial) is that systemic administration may not achieve the same high local concentration of the thrombolytic agent within the ischemic territory. Furthermore, IV delivery relies more heavily on the patency of collateral circulation to reach the distal area. By reserving 25% of the total tPA dose for post-recanalization administration, we aimed to enhance distal reperfusion while minimizing the bleeding risk associated with full-dose systemic thrombolysis. The favorable PBV findings in this study support the potential efficacy of this modified protocol.

Importantly, the potential efficacy of IV rather than intra-arterial administration may depend on the degree of collateral circulation. Well-developed collaterals could allow tPA to reach hypoperfused tissue even before complete recanalization, whereas patients with poor collateral flow might derive greater benefit from post-thrombectomy IV tPA, as drug delivery to the ischemic territory would be more effective once macrovascular flow has been restored. This interaction between collateral status and the benefit of adjunctive tPA warrants further investigation.

To further explore safety considerations, we conducted a subgroup analysis focusing on factors associated with hemorrhagic complications. Previous studies have suggested that hyperperfusion may be a potential risk factor for hemorrhagic complications (12). However, our findings indicate a more nuanced relationship between tissue perfusion and safety outcomes. In our study, an adequate increase in PBV after thrombectomy appeared to be associated with improved functional outcomes, whereas patients who failed to demonstrate a sufficient rise in PBV were more likely to experience ICH. These results suggest that inadequate microvascular reperfusion, rather than hyperperfusion itself, may predispose patients to hemorrhagic transformation, underscoring the importance of achieving balanced and effective tissue-level reperfusion. Reported rates of ICH after combined IV tPA and mechanical thrombectomy range from approximately 9 to 16.3% in large randomized trials such as SWIFT PRIME and DIRECT-MT (15, 16). Although our study did not find a significant difference in hemorrhagic complications between the two groups, the incidence of such events in the post-IV tPA group was 14.3%, warranting careful consideration when adopting this approach. This relatively high rate may be partly attributable to the higher mean age of our cohort compared with previous studies and the longer O2R time.

However, the potential detrimental effects of additional exogenous tPA administration must be carefully considered. Recent experimental studies have highlighted the “tPA paradox,” demonstrating that tPA exerts non-fibrinolytic neurotoxic roles in the ischemic brain following reperfusion (17). Specifically, extravasated tPA can exacerbate ischemic injury by promoting the activation of matrix metalloproteinases (particularly MMP-9), leading to blood–brain barrier (BBB) disruption, neuroinflammation, and an increased risk of hemorrhagic transformation (18). Recognizing these dose-dependent neurotoxic risks, our institution strictly designed the 75–25% protocol to preserve the standard total cumulative dose. By avoiding an excess total dose of exogenous tPA, we aimed to harness its fibrinolytic benefits for microvascular reperfusion while mitigating the risk of tPA-mediated BBB disruption and neurotoxicity.

Several limitations to this study need to be kept in mind. First, the retrospective design of this single-center study inherently introduces selection bias. As the administration of adjunctive post-IV tPA was based on the evolution of our institutional treatment protocol over time, rather than randomization, the treatment cohorts are subject to historical or chronological bias. Consequently, patients in the post-IV tPA group may have been treated during a later period compared to the standard method group. This chronological discrepancy introduces potential unmeasured confounders, such as interim advancements in thrombectomy devices and increased operator experience, all of which might have independently contributed to the improved outcomes observed in the post-IV tPA group. Second, the optimal timing and dose of post-EVT IV tPA remain to be determined, and the mechanisms by which tPA may enhance microvascular reperfusion were not directly evaluated in this study. In addition, PBV was measured 15 min after the administration of adjunctive IV tPA. Whether this early time point adequately reflects the full pharmacological effect on microvascular reperfusion is uncertain. Future studies incorporating delayed imaging modalities, such as single-photon emission CT or perfusion magnetic resonance imaging the following day, will be essential to clarify the relationship between immediate PBV changes and sustained improvements in cerebral perfusion. Third, the sample size of our study, particularly in the post-IV tPA group (n = 21), is small. This limitation substantially reduces the statistical power, increases the risk of type II error, and restricts the generalizability of our findings. Due to this limited cohort size, extensive multivariable adjustments or complex matching were not performed to avoid the risk of model overfitting and unstable effect estimates, which underscores the exploratory nature of this study. Finally, grouping patients with mTICI 2b and mTICI 3 into a single successful reperfusion cohort introduces potential heterogeneity. Although both grades represent successful macrovascular recanalization, the broad reperfusion range inherent to mTICI 2b may differentially impact PBV values and functional outcomes compared to complete reperfusion (mTICI 3). Given the limited sample size, a meaningful subgroup analysis stratifying these angiographic grades could not be performed. Ultimately, prospective, multicenter, randomized controlled trials are warranted to confirm the efficacy and safety of adjunctive IV tPA following successful EVT.

## Conclusion

Adjunctive IV tPA following successful EVT may enhance microvascular reperfusion, as indicated by increased PBV, and contribute to improved functional outcomes. However, the occurrence of hemorrhagic complications, particularly among older patients and those with prolonged O2R times, underscores the need for cautious patient selection and further prospective validation. Importantly, given the retrospective and non-randomized design of our study, these findings are primarily hypothesis. Further prospective, randomized comparative studies are strongly warranted to confirm the efficacy and safety of this approach.

## Data Availability

The raw data supporting the conclusions of this article will be made available by the authors, without undue reservation.

## Supplementary material

SUPPLEMENTARY FIGURE S1 Comparison of parenchymal blood volume (PBV) parameters according to DWI lesion growth. Patients with DWI lesion growth show significantly lower post-EVT PBV **(A)** and ΔPBV ratios **(B)** compared to those without lesion growth (*p* = 0.002 and *p* = 0.0134, respectively).
