# Supplementary figures and images for: Safety and efficacy of intravenous tPA after successful thrombectomy for large vessel occlusion: a retrospective study
Source: Front Neurol. 2026 May 29;17:1793073. doi: 10.3389/fneur.2026.1793073 (PMC13260885; doi:10.3389/fneur.2026.1793073)

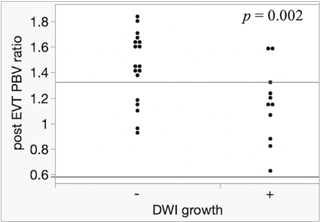

Supplement: SUPPLEMENTARY FIGURE S1 — Comparison of parenchymal blood volume (PBV) parameters according to DWI lesion growth. Patients with DWI lesion growth show significantly lower post-EVT PBV (A) and ΔPBV ratios (B) compared to those without lesion growth (p = 0.002 and p = 0.0134, respectively). [file Image_1.tiff]

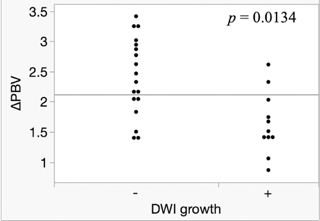

Supplement: Supplementary file 2 [file Image_2.tiff]
